# Evaluation of *in vitro* and *in vivo* release of recombinant human nerve growth factor from bioengineered human stromal lenticule

**DOI:** 10.3389/fbioe.2025.1588629

**Published:** 2025-09-19

**Authors:** Molly Tzu-Yu Lin, Yu-Chi Liu, Isabelle Xin Yu Lee, Domitilla Mandatori, Nicola Detta, Jing Ying Evelina Han, Letizia Pelusi, Leonardo Mastropasqua, Mario Nubile, Franca Cattani, Tiziana Romeo, Marcello Allegretti, Assunta Pandolfi, Harminder S. Dua, Jodhbir S. Mehta

**Affiliations:** ^1^ Tissue Engineering and Cell Therapy Group, Singapore Eye Research Institute, Singapore, Singapore; ^2^ Department of Cornea and External Eye Disease, Singapore National Eye Centre, Singapore, Singapore; ^3^ Department of Medical, Oral and Biotechnological Sciences, Center for Advanced Studies and Technology (De Castro et al.), StemTeCh Group, “G. d’Annunzio” University of Chieti-Pescara, Chieti, Italy; ^4^ Dompé farmaceutici SpA, Via Tommaso de Amicis, Naples, Italy; ^5^ Department of Medicine and Aging Science, “G. d’Annunzio” of Chieti-Pescara, Chieti, Italy; ^6^ Ophthalmology Clinic, Department of Medicine and Aging Science, “G. d’Annunzio” of Chieti-Pescara, Chieti, Italy; ^7^ Dompé farmaceutici SpA, Via Campo di Pile, L’Aquila, Italy; ^8^ Department of Cornea and External Disease, Nottingham Hospital, Nottingham, United Kingdom

**Keywords:** nerve growth factor, corneal stromal lenticules, decellularization, tissue engineering and regenerative medicine, nerve regeneration, drug delivery, microparticles (MPs)

## Abstract

**Introduction:**

Small incision lenticule extraction (SMILE)-derived lenticules have been repurposed as biocompatible scaffolds to incorporate and release therapeutic substances for ocular therapeutics. We aim to investigate the *in vitro* and *in vivo* release profiles of recombinant human nerve growth factor (rhNGF) from bioengineered human stromal lenticules prepared with microparticles incorporated with rhNGF (rhNGF-MPs) for up to 1 and 4 weeks, respectively.

**Methods:**

Upon bioengineered lenticule implantation, slit lamp, Anterior Segment Optical Coherence Tomography, and *In Vivo* Confocal Microscopy were performed to assess corneal biocompatibility, central corneal thickness (CCT), corneal nerve -fiber density (CNFD), -branch density, and -fiber length. Rabbit cornea, tears and aqueous humour were collected to quantify rhNGF release *in vivo*.

**Results:**

A rapid *in vitro* release of rhNGF was detected until day 2, and with sustained release over 7 days. The pattern remains comparable even with the presence of antibiotic-antimycotic, trypan blue or fluorescein. Throughout the *in vivo* follow-up, no signs of corneal haze, edema, infiltration, or pathological increase in CCT were observed. A significant increase in CNFD (p = 0.001) at week 4 was reported in rhNGF-MPs than in Blank-MPs group. Finally, significantly higher NGF content in rabbit cornea and tear was found in rhNGF-MPs group compared to the endogenous NC group (p = 0.035 and p = 0.043, respectively).

**Discussion:**

Bioengineered lenticules exhibit sustained rhNGF release for at least 7 days *in vitro* and up to 1 month *in vivo*. These results, together with absence of adverse effects, and significant increase in CNFD at 4 weeks after lenticule implantation suggest its promising potential for clinical use.

## Introduction

The cornea is an avascular tissue situated at the outmost layer of the eye. Having high transparency with optimal refractivity is essential to permit proper light transmission for clear vision. The densely innervated feature of human cornea (approximately 7,000 sensory nerves per mm^2^) (Müller et al., 2003) has signified the importance of corneal nerves in facilitating sensory-dependent corneal and tearing reflexes (Marfurt et al., 2010). Numerous studies have supported the significance of corneal nerves in sustaining homeostasis and a healthy ocular surface through providing trophic support to various cells such as secreting neuromediators to promote corneal wound healing (Müller et al., 2003; Shaheen et al., 2014; Belmonte et al., 1999).

The corneal nerve dysfunction following traumatic injury, viral infection, surgical intervention (Bandeira et al., 2019), or systemic diseases (Mansoor et al., 2020; So et al., 2022) significantly hinders the corneal epithelium renewal and healing abilities. These pathological conditions when left unattended, could progress further to the development of neurotrophic keratopathy (NK). The devastating hallmark of NK displays diminished or complete loss of corneal sensitivity with poor epithelial healing rate. Its manifestation without appropriate therapeutic intervention could consequently cause corneal ulcer and may result in visual impairment and potentially permanent blindness (Bonini et al., 2003; Sacchetti and Lambiase, 2014).

Nerve growth factor (NGF) is a soluble protein and a prominent member of the neurotrophin family. Many studies have reported its effective role in promoting the repair of injured nerves and corneal re-innervation *in vivo* (Wan et al., 2021; Yam et al., 2022; Savignat et al., 2007). It also exhibited the ability to stimulate the growth of corneal epithelial cells as well as to sustain limbal epithelial stem cell potential (Li et al., 2012; Park et al., 2016; Yang et al., 2021). On this basis, recombinant human NGF (rhNGF) was produced in *Escherichia Coli* for topical use. A recent study of an 8-week topical rhNGF treatment regime has shown to improve tear film secretion, corneal sensitivity and promote nerve fiber regeneration in NK patients with a persistent epithelial defect or corneal ulcer (Mastropasqua et al., 2020). Besides, rhNGF eye drops were reported to effectively alleviate the symptoms and signs in patients with moderate to severe dry eye disease in a phase IIa, prospective, open label, multiple-dose study (Sacchetti et al., 2020). The promising therapeutic results in clinical trials with the effects of stimulation of corneal wound healing, improvement in corneal sensitivity, corneal nerve morphologies, as well as damage in ocular surface have signified the profound contributions of NGF in the treatment of ocular surface diseases (Bonini et al., 2018; Sacchetti et al., 2020; Mastropasqua et al., 2020). However, the eye drop formulation was limited by its high costs and requirement of low temperature storage condition. The challenging frequent instillation of up to six times per day with uncomfortable side effects can lead to poor compliance, reducing ocular bioavailability with undesirable treatment efficiency (Pflugfelder et al., 2020).

The potential use of human stromal lenticules derived from small incision lenticule extraction (SMILE) procedure, a femtosecond laser-based refractive treatment of myopia, as an ocular drug delivery system has been suggested as one of the practical solutions for therapeutic treatment for corneal diseases (Santra et al., 2022; Mastropasqua et al., 2022). The natural source of collagen-rich extracellular matrix (ECM) present in the corneal stromal layer, together with its characteristics of having high mechanical strength, transparency and low immunogenicity (Zhang et al., 2017), have put forth a repurpose horizon of stromal lenticule in the pharmaceutical and regenerative therapy (Santra et al., 2022) in clinical ophthalmology, which exceed far beyond its ordinary fate of being discarded. While increasing numbers of studies are using SMILE-derived human stromal lenticules as therapeutic depot for cell delivery (Hong et al., 2018; Aghamollaei et al., 2021) or as surgical patch graft for structural restoration (Pant et al., 2020; Yang et al., 2020), there are currently no study employing bioengineered SMILE-derived lenticules with rhNGF-loaded PLGA-microparticles (rhNGF-MPs) in the *in vivo* system. This study therefore provides valuable insights for assessing its potential translational applications to address the limitations of clinically available rhNGF eye drop formulation.

**FIGURE 1:**
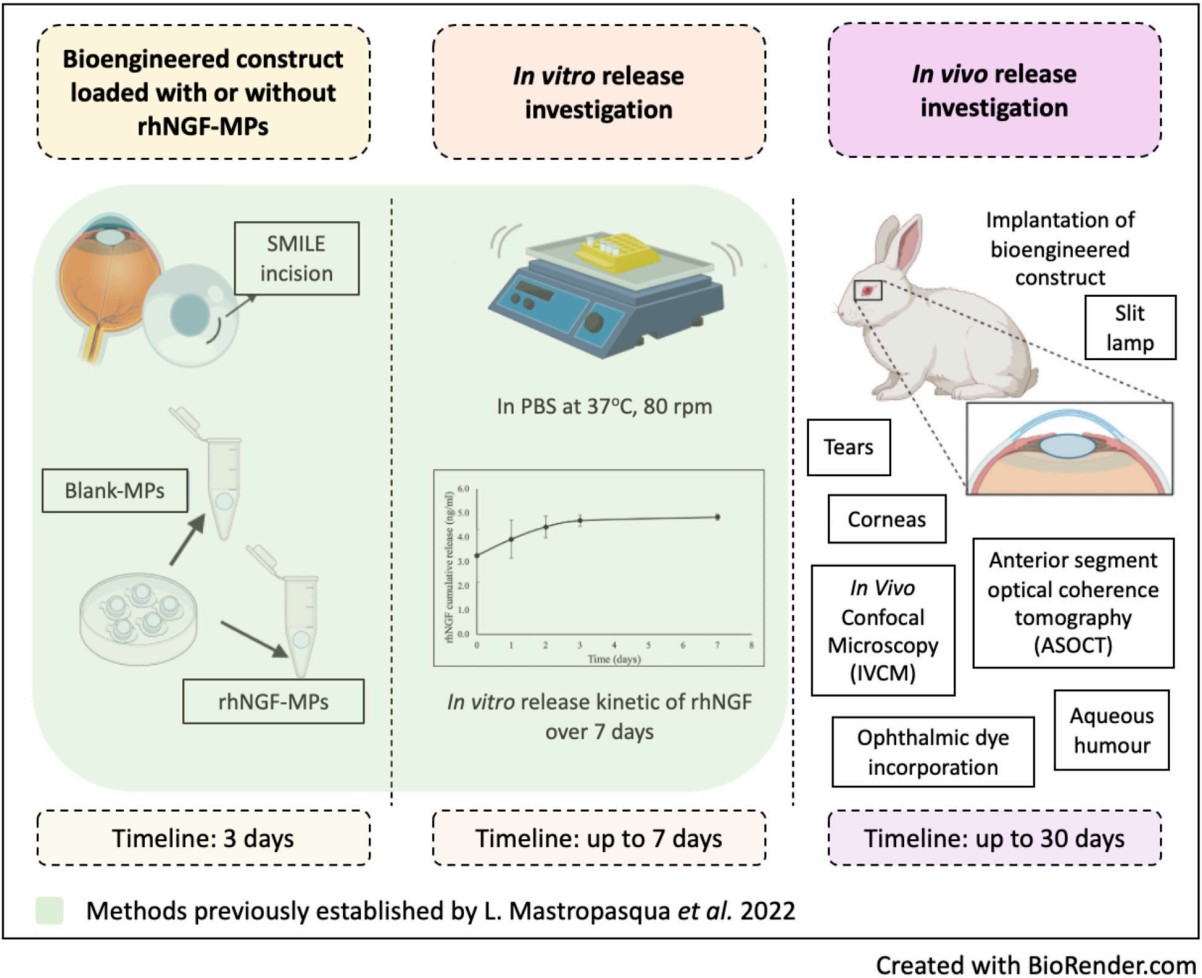
Schematic diagram outlines the methods implemented from previous and current study. Created in BioRender. Liu, Y. (2025) https://BioRender.com/cnoesyb.

Incorporating therapeutic substances in an appropriate vehicle, followed by loading it in the bioengineered scaffold for re-implantation back to corneal stromal layer, could enhance the bioavailability and biodistribution of therapeutic molecules by prolonging its corneal residence time, which is especially beneficial for corneal therapy as targeted drug delivery. A previous *in vitro* proof-of-concept study published by our group has successfully incorporated rhNGF-MPs into the SMILE-derived decellularized lenticules with preserved biological activities *in vitro* (Mastropasqua et al., 2022) (Figure 1). The evident indication of sustained *in vitro* release of rhNGF from decellularized human lenticules for up to a month has showed its promising potential of using corneal stromal lenticule as a depot for incorporating active pharmaceutical ingredient for therapeutic treatment of ocular diseases.

As a further study from Mastropasqua et al. (2022), the present study focuses on moving forward the established method of *in vitro* sustained release profile to *in vivo* study to further investigate its translational potential for future clinical applications. The overlay of the *in vitro* analysis included in this study serves as a method validation to ensure the establishment of similar *in vitro* release profile prior to the main focus of this study–the *in vivo* evaluations. In consideration of the translational prospect of re-implanting the rhNGF-loaded lenticules and potential challenges encountered during the surgery, it is essential to perform a multifaceted investigation to pave a smooth clinical transplant procedure with minimal risk of infection. Including antibacterial and antifungal agents in the lenticules loaded with therapeutic agents could further minimize the potential risk of infection caused during surgical procedures. On the other hand, the option to incorporate safe-to-use ocular dyes could be a great help to facilitate surgeons for better visualization during surgery. However, to the best of our knowledge, the compatibility between rhNGF-MPs and antimicrobial agents, fluorescein or trypan blue remains unclear.

Development of novel drug and its effective therapeutic strategies for ocular disease treatment requires an experimental animal model that shares similar etiology and pathology (Zernii et al., 2016). Although rodent models are commonly employed in experimental visual research, their small eyeball size has greatly limited the access of clinical manipulations. Besides, the significant differences in pharmacokinetics and the required therapeutic dosages make them inadequate models for pre-clinical efficacy and safety studies (Short, 2008). In particular, they are poorly suitable to be used for testing novel drug delivery approaches, such as controlled release of pharmaceuticals using intra-ocular implants, or drug-eluting intraocular lenses (Morrison and Khutoryanskiy, 2014). On the other hand, non-human primates, although exhibiting clinical presentation closely resembling those seen in humans, special laboratory facilities and high costs impeded the experimental options (Zernii et al., 2016). In contrast, rabbits are cost-effective and relatively easy to handle. Apart from greater similarity in ocular anatomical, biochemistry and biomechanical features to humans than rodents, the larger ocular dimensions, similar optical system, as well as conjunctival cavity volume make rabbit eye suitable for various surgical manipulations, including corneal transplantation and laser refractive procedures (Zernii et al., 2016; York and Steiling, 1998). It’s also worth mentioning that the kinetics of intraocular drug penetration follows the same pattern in rabbit and human eyes (Mishima, 1981). Moreover, it was also found that human and rabbits share similar three-dimensional organization of stromal lamellae, three-dimensional keratocyte features (Ojeda et al., 2001), and corneal wound healing response (Miyamoto et al., 2003; Torricelli et al., 2015; Santhanam et al., 2017). With these abovementioned features, rabbits are highly suitable for evaluating safety and efficacy of novel drug and investigating its effectiveness of therapeutic approaches.

In this study, we aim to 1) validate the method by performing the same procedures of lenticule preparation and *in vitro* release as previously described (Mastropasqua et al., 2022), as well as 2) to further investigate the *in vivo* release profile of rhNGF at 4 h, day 2, 1-week, 2-week and 4-week after performing *in vivo* implantation procedure in rabbit cornea. Moreover, multiple clinical examinations using slit lamp, anterior segment optical coherence tomography (ASOCT), and. *In Vivo* Confocal Microscopy (IVCM) were carried out to thoroughly evaluate the corneal biocompatibility, lenticule placement, and the potential capability of corneal nerve regeneration, respectively. In addition, we have also investigated the *in vitro* release profile of rhNGF after exposure to antimicrobial reagent, trypan blue and fluorescein to evaluate its potential clinical application for lenticule implantation.

## Materials and methods

### Ethical statement

The lenticules used for *in vitro* experiments were obtained from patients who underwent refractive surgery with SMILE procedures at the Singapore National Eye Centre, Singapore. Patients with manifest refraction spherical equivalent range −4.00 to −6.00 D, with astigmatism less than 0.75 D were included in the study. All patients were above the age of 18 and had a normal corneal topographic pattern and stable refraction for at least 1 year preoperatively. Patients who had any ocular pathology other than refractive error or history of ocular surgery were not included. The study was approved by the institutional review board of SingHealth, Singapore (CIRB/109/A) and conducted in accordance with the Declaration of Helsinki with informed consent form obtained from all patients.

For *in vivo* studies, research-grade human cadaver corneal tissues were procured from Lions Eye Institute for Transplant and Research (Tampa, FL, USA) with written consent from the donor’s next-of-kin to be used for research. Donors who received any refractive surgery were excluded in the study. The corneoscleral donor tissues were preserved in Optisol-GS (Bausch & Lomb, Rochester, NY, USA) at 4 °C until further process for *in vivo* experiments. All research work performed using human-derived tissue was carried out in accordance with the declaration of Helsinki.

### Surgical procedures for lenticule extraction

Corneal stromal lenticules used in the *in vitro* studies were obtained during the SMILE procedure using a 500 kHz VisuMax system (Carl Zeiss Meditec AG, Jena, Germany) as described previously (Liu et al., 2017a). The lenticules used for *in vivo* studies were obtained the same surgical procedure using human cadaveric cornea by two experienced surgeons (JSM and YCL). Briefly, the corneoscleral buttons were mounted with Coronet artificial chambers. The following femtosecond laser parameters were used: 6.5-mm optical zone, 7.9-mm cap diameter, 120-μm cap thickness. According to the Visumax software, a correction power set at −6.5D generated a biconvex lenticule with a minimum thickness at 15-μm at the peripheral edge and maximum thickness of 120-μm at the center. The extracted stromal lenticules (n = 24) were transferred into wash buffer containing Opti-Mem-I solution, human endothelium serum-free medium, Dulbecco’s phosphate-buffered solution (DPBS 1 × , calcium and magnesium), 25 μ g/mL of Gentamincin solution, 100 U/mL of Penicillin/Streptomycin solution, 750 mg/mL of Amphotericin B for rinsing and stored in glycerol (Sigma-Aldrich, St. Louis, MO, USA) at −80 °C (Liu et al., 2017b) until further experiments. All reagents used were purchased from Gibco^®^ by Life Technologies (Waltham, MA, USA) unless otherwise specified.

### Preparation of MPs loaded with rhNGF

The preparation of PLGA-MPs loaded with rhNGF was performed as described previously (Mastropasqua et al., 2022). The microparticles were produced using a “customized” double emulsion with solvent evaporation method with averaged size of 5 μ m, and encapsulation efficiency of more than 55%.

### Decellularization treatment

The SMILE-derived lenticules were decellularized as described previously (Yam et al., 2016). Briefly, the lenticules were thawed, rinsed thoroughly in 15 mL of DPBS 1 × (No calcium, no magnesium), for a total of 4 times and decellularized by incubating in 1 mL of 0.1% SDS solution for 24 h under 300 rpm agitation at room temperature (RT). Following three washes in PBS 1 × (24 h each), decellularized stromal lenticules were ready to be engineered with rhNGF-MPs.

### Lenticules engineered with rhNGF-MPs

The decellularized stromal lenticules were further dehydrated for 2 h at 60 °C, and immediately engineered with PLGA-MPs loaded with rhNGF by incubating in rhNGF-MPs suspension (5 mg in 0.175 mL of 0.9% NaCl) as described in a previous study (Mastropasqua et al., 2022). After 3 h of incubation with constant shaking at 200 rpm at RT, lenticules were removed from the rhNGF-MPs suspension and washed 10 times or 2 times in 0.4 mL of 0.9% NaCl before *in vitro* and *in vivo* release, respectively. For experimental control group, stromal lenticules were subjected PLGA-MPs suspension without incorporating rhNGF (Blank-MPs).

### *In vitro* kinetic release of rhNGF from decellularized lenticules

The *in vitro* release profiles of rhNGF are evaluated as previously described (Mastropasqua et al., 2022). Specifically, lenticule engineered with rhNGF-MPs (n = 6) were transferred in 0.5 mL polypropylene Eppendorf protein LoBind tubes containing 150 μ L of 0.9% NaCl and incubated at 37 °C with orbital shaking at 80 rpm. The samples were collected at each time point for the analysis of *in vitro* rhNGF release: 5 min, 2 h, 4 h, day 2, day 3, and day 7. At each time point, each lenticule was transferred into a new tube containing 150 μ L of 0.9% NaCl and continued to incubate at 37 °C at 80 rpm. The collected samples at each time point were stored immediately at −20 °C for the ELISA analysis for detection of rhNGF concentration.

To investigate the effect of antibiotic-antimycotic (anti-anti) on the *in vitro* release profiles of rhNGF, lenticules engineered with rhNGF-MPs were washed in 0.9% NaCl containing anti-anti (1 × ) for 10 times. To evaluate the potential of using ocular dyes as visually assistive tool to implant rhNGF-containing lenticule into corneal stroma, rhNGF-MPs loaded lenticules was firstly incorporated with either fluorescein or trypan blue to examine the *in vitro* release profile. Following the rhNGF-MPs loading procedure as previously described, the excessive rhNGF-MPs were first rinsed off in 0.9% NaCl for 9 times, followed by briefly immersing the lenticules in either trypan blue (Vision Blue, Dutch Ophthalmic USA) or 2% w/v fluorescein solution (MINIMS^®^; Bausch & Lomb, Australia) once (1 s) immediately before the *in vitro* release. The samples collection and storage were carried out the same way as described at 5 min, 2 h, 4 h, day 2, and day 3.

### *In Vivo* kinetic release of rhNGF from decellularized lenticules

#### Animals

Male New Zealand white rabbits (2.8–3 kg, age 3–6 months) used in this study were purchased from Envigo (Isarael). Animals were housed under standard conditions with food and water provided *ad libitum* in a light-controlled room and set temperature and humidity. All animal care and experimental procedures were carried out in accordance with tenets of the Association for Research in Vision and Ophthalmology Statement for the use of Animal in Ophthalmic and Vision Research, and the protocol was approved by the Institutional Animal Care and Use Committee of SingHealth. No. (2019/SHS/1511).

#### Sample size rationale statement

The sample size was designed based on group-specific treatment strategies and ethical considerations to ensure sufficient statistical power while minimizing animal use following the 3R principle (Replacement, Reduction and Refinement). Our experimental groups (rhNGF-MPs and Blank-MPs) adopt the independent eye strategy to ensure no cross-over effects from the treatment while the control groups implement the paired design with both eyes subjected to the same control conditions. The required sample size was calculated based on the pilot data of the primary outcome, which was corneal nerve fiber density (CNFD), from 2 mice (4 eyes). The mean CNFD was 18.2 ± 2.0 fibers/mm^2^ and 13.1 ± 2.0 fibers/mm^2^ at week 4 for the rhNGF and blank group, respectively. Hence, a sample size of 4 eyes, with a power of 80% and at a 5% level of significance, was sufficient to detect the difference between rhNGF-MPs treated and untreated control group.

#### *In vivo* implantation of bioengineered rhNGF-MPs loaded lenticule

A total of 30 rabbits were randomly allocated into four groups: rhNGF-MPs (5 mg/mL; n = 17 eyes), blank-MPs (5 mg/mL; n = 17 eyes), positive control (PC; intrastromal pocket created without lenticule implantation; n = 12 eyes), and negative control (NC; untreated control; n = 14 eyes). A 7.5 mm intrastromal pocket was created by the Visumax femtosecond laser in rabbit eyes from PC, blank-MPs, and rhNGF-MPs groups at the depth of 120 μm and over the pupillary center (Liu et al., 2018). The lenticules with and without rhNGF-MPs were implanted and distended into the intrastromal pocket by a single surgeon (JSM) (Liu et al., 2018). Following the implantation procedures, the pocket incision was sutured with two stiches using a 10-0 nylon suture (Johnson & Johnson, New Brunswick, NJ) with triple knot. Subsequently, subconjunctival injection with 4 mg gentamicin sulfate and 0.4 mg dexamethasone sodium phosphate was given to minimize the inflammation. To minimize the potential differences between groups, sutures were also performed on the PC group after intrastromal pocket creation. Rabbit eyes without any surgery procedure served as negative control for endogenous NGF detection. Following lenticule implantation, all eyes received a postoperative regime of TobraDex^®^ eye drop (Santen, Emeryville, California, USA) four times a day until the sacrifice time point at 4 h, day 2, day 7, week 2 and week 4 after the procedures.

#### Evaluation of corneal biocompatibility

A topical application of 0.9% sodium chloride solution was given prior to acquire the slit lamp images. The images were taken by a slit lamp biomicroscope with white light (LED Slit Lamp MW50D, Righton, Tokyo, Japan) at each time point to examine the overall status of the cornea at the site of implantation.

#### Evaluation of central corneal thickness

Three high-resolution ASOCT (RTVue; Optovue, Inc., Fremont, CA, USA) corneal cross-sectional scans were captured at each time point (4 h, day 2, day 7, week 2, week 3, and week 4 after lenticule implantation) to evaluate the changes in the central corneal thickness (CCT), as well as to observe the lenticule placement and the potential pathological thickening of the cornea due to the surgical procedures. The CCT was measured using an in-built scale with the unit of μm (Liu et al., 2014).

#### Evaluation of corneal nerve regeneration

The potential of nerve regeneration resulting from the released rhNGF-MPs was evaluated using *in vivo* confocal microscopy (IVCM, HRT3; Heidelberg Engineering GmbH, Heidelberg, Germany) before (baseline) and after (4 h, day 2, day 7, week 2, week 3, week 4) lenticule implantation. Carbomer gel (Vidisic^®^; Mann Pharma, Berlin, Germany) was applied on the confocal lens as the immersion fluid before each procedure. Five anatomical areas (central, superior, inferior, nasal and temporal cornea) were imaged during each scan. For the corneal nerve analysis, 10–15 most representative and best-focused images of subbasal nerves were selected from each area, and were further analyzed using CCMetrics software (University of Manchester, Manchester, UK) after manual annotations (Liu et al., 2021a). The following three parameters were assessed for further interpretations: corneal nerve fiber density (CNFD, number of fibers/mm^2^); corneal nerve branch density (CNBD), computed as the number of branch points on the main fibers/mm^2^; and corneal nerve fiber length (CNFL), calculated as the total length of fibers (mm)/mm^2^.

#### NGF levels in tears and aqueous humor

Tears and aqueous humor collection were carried out at 4 h, day 2, day 7, week 2, and week 4 after implantation. Before the procedure, sedation was given through intramuscular injection of 40 mg/kg of ketamine hydrochloride (Parnell Laboratories, new South Wales, Australia) and 4 mg/kg of xylazil-20 hydrochloride (Troy Laboratories, New South Wales, Australia). Tears were collected by placing a Schirmer strip onto the cul-de-sac of the semi-sedated rabbit for 5 minutes without using topical anesthesia (Yawata et al., 2016). An approximately 200–250 μL of aqueous humor was collected using a 27-gauge needle. All samples were collected in Eppendorf Protein LoBind tubes and were stored immediately at −80 °C until ELISA analysis. To elute the tear proteins, the wetted part of each Schirmer strip was first cut into small pieces and submerged in 300 μL of ice-cold lysis buffer of 50 mM Na_2_HPO_4_ (pH 7.4) containing 5 μg/mL of Aprotinin, Leupeptin, Pepstatin A and 1 mM phenylmethylsulfonyl fluoride (Liu et al., 2021b). Stainless steel beads (diameter of 2.4 mm; Omni International, Inc., Georgia, USA) were added into each sample to facilitate the homogenization using an Omni Bead Ruptor 24 tissue homogenizer (Omni) at 2.75 m/s for 20s at 4 °C. The homogenization step was repeated for a total of 3 times, each with 5 min break on ice. Subsequently, the samples were centrifuged at 20,000 ×g at 4 °C. Eventually, the supernatant was collected and stored at −80 °C until ELISA analysis. ELISA procedure was performed according to the manufacturer’s instructions using commercially available kits for the measurement of NGF concentrations (RayBiotech Life Inc.; GA, USA). The optical density (OD) was determined at 450 nm using a Tecan Infinite M200 (Tecan, Männedorf, Switzerland). All samples were run without dilution in duplicate. All data was normalized to endogenous NGF detected in NC group and expressed as fold of NC. Tear NGF level was expressed as pg per milliliter per millimeter of the Schirmer wet length after normalized to the Schirmer wet length.

#### NGF levels in corneas

Rabbits were euthanized with overdose of pentobarbitone through cardiac injection (300 mg/kg) at each time point (4 h, day 2, day 7, week 2, and week 4). Each rabbit cornea was excised carefully and collected in an Eppendorf Protein LoBind tube. The weight of the excised corneas was recorded and stored immediately at −80 °C. Prior to homogenization, tissues were thawed and cut into small pieces with size of approximately 3 mm × 3 mm. Cornea tissues were lysed by adding 350 μL of lysis buffer and the cornea tissue lysate was collected the same way as described for the elution of tear proteins. ELISA analysis was performed as described and the NGF content per corneal tissue weight (pg/g) was calculated in each sample before normalizing the data to endogenous NGF (NC group) for comparison.

#### Statistical analysis

All data were expressed as mean ± standard deviation (SD). Statistical comparisons for the *in vivo* studies among different groups (with respect to NC group) and time points (week 4 versus baseline) were performed using the Mann-Whitney U test and Wilcoxon test, respectively (GraphPad Prism 8 Software). The level of NGF detected in each sample using ELISA was interpolated from the standard curve using four-parameter logistic model using a GraphPad Prism 8 software (SanDiego, CA, USA). A *p*-value <0.05 was considered statistically significant.

## Results

### *In vitro* kinetic release profiles of rhNGF from MPs loaded in decellularized lenticules

**FIGURE 2:**
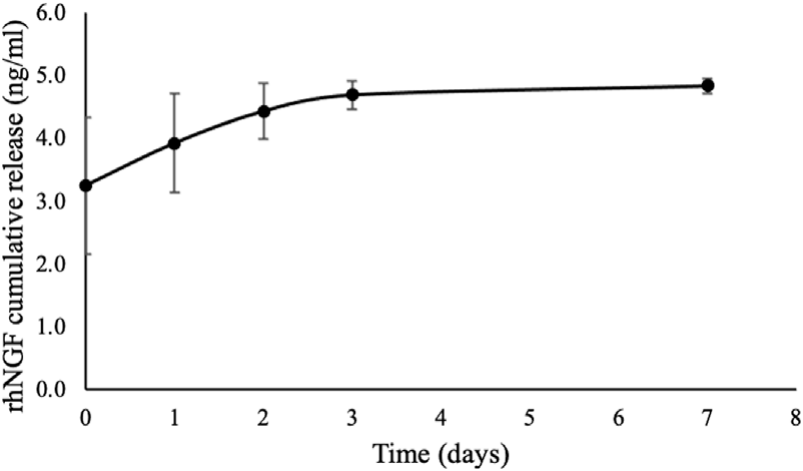
*In vitro* release kinetic of rhNGF-MPs-loaded SMILE-derived lenticules with similar donor age and thickness (n = 4). The *in vitro* release of rhNGF was collected at 5 min (day 0), 2 hrs and 4 hrs (day 1), day 2, day 3, and day 7 followed by ELISA analysis. The graph depicts averaged rhNGF cumulative release (ng/mL) ± SD, showing a burst release of rhNGF from the MPs loaded to decellularized lenticule, followed by a sustained release up to 7 days *in vitro*.

There was an initial burst of 3.25 ± 1.09 ng/mL rhNGF release at the first 5 minutes from the decellularized lenticules previously loaded with rhNGF-MPs. A rapid *in vitro* release of rhNGF was observed at cumulative release of 4.44 ± 0.44 ng/mL until day 2 (around 93%), followed by sustained release at the cumulative concentration of 4.69 ± 0.22 ng/mL (around 4%) to 4.84 ± 0.12 ng/mL (around 2%) for 1 week (Figure 2). The kinetic analyses of *in vitro* release profile showed a rapid release of rhNGF during the first 2 days but remained steady release throughout the study period.

**FIGURE 3:**
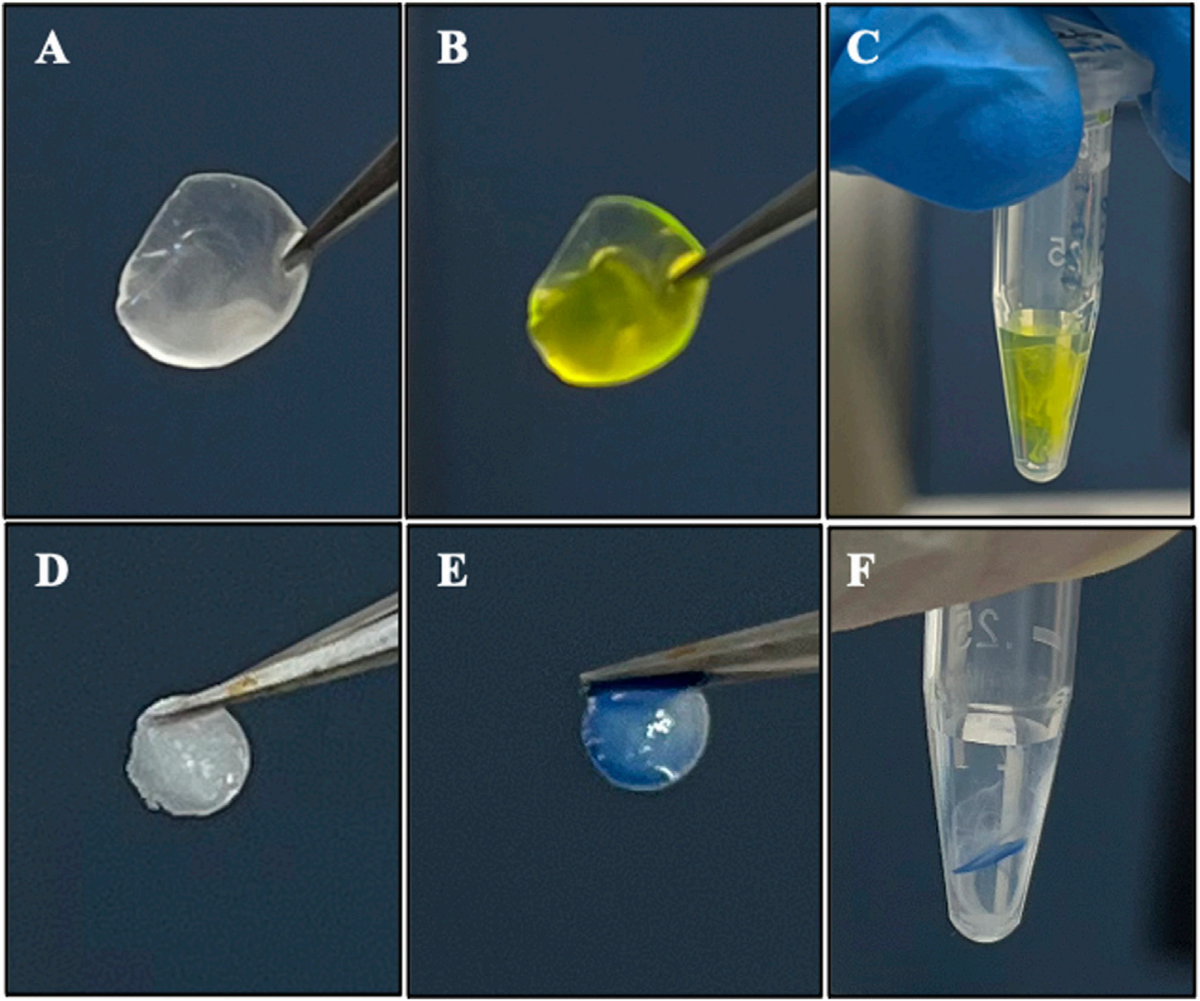
Representative images of exposing rhNGF-MPs loaded decellularized lenticules to **(A–C)** fluorescein (2% w/v) and **(D–F)** trypan blue solution for *in vitro* release. **(A,D)** After 3 h of loading procedure, the lenticules were washed in 0.9% NaCl for 9 times before **(B)** dipping it once in the fluorescein and **(E)** trypan blue solution **(C,F)** *in vitro* release of the rhNGF-MPs loaded lenticules in 120 μL of 0.9% NaCl after fluorescein and trypan blue staining. The samples were collected at 5 min (day 0), 2 h and 4 h (day 1), day 2, and day 3 for ELISA analysis.

To confirm the compatibility of using fluorescein or trypan blue as potential visually assistive tool to implant rhNGF-containing lenticule into corneal stroma, we have investigated the *in vitro* release profile of rhNGF after short exposure to fluorescein or trypan blue dyes. Figure 3 shows the before and after immersing in dyes (Figures 3A–D) shortly before the *in vitro* release in 0.9% NaCl (Figures 3E,F).

**FIGURE 4:**
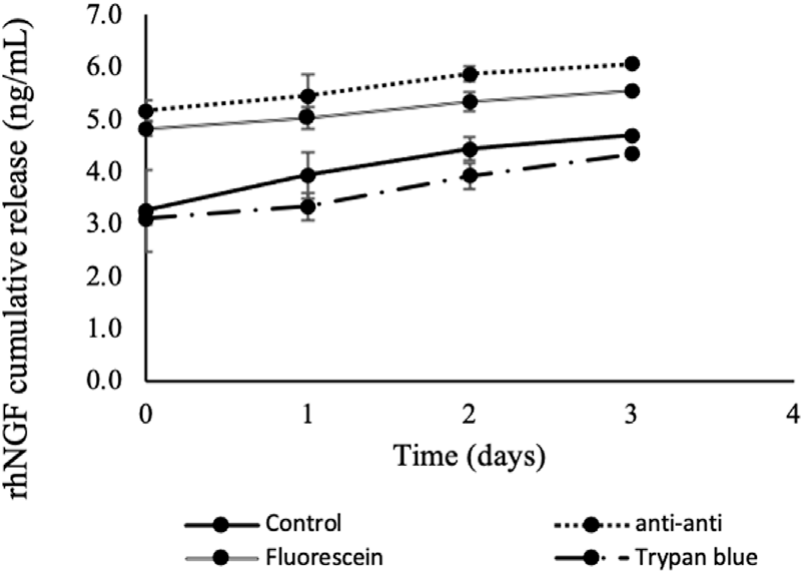
*In vitro* cumulative release kinetic of rhNGF-MPs-loaded lenticules. Decellularized SMILE-derived lenticules were first loaded with rhNGF-MPs before undergoing 4 washing treatments (n = 4/treatment group): antibiotic-antimycotic (1 × ), quick staining in fluorescein, trypan blue solution and controls (only in 0.9% NaCl). The *in vitro* release of rhNGF after respective washing was collected at 5 min (day 0), 2hrs and 4hrs (day 1), day 2 and day 3 followed by ELISA analysis. The graph depicts averaged rhNGF cumulative release (ng/mL) ± SD over 3 days upon various washing treatments.

The *in vitro* release profiles of rhNGF showed increasing rhNGF cumulative release from 5.17 ± 1.02 to 6.06 ± 0.15 ng/mL after exposure to anti-anti (1 × ), 4.82 ± 0.93 to 5.56 ± 0.18 ng/mL and 3.10 ± 1.85 to 4.34 ± 0.25 ng/mL after fluorescein and trypan blue treatment, respectively (Figure 4). The release profiles from control group treated without ocular dye or antimicrobial agents (washed only with 0.9% NaCl) also showed increasing release of rhNGF over time, with cumulative release from 3.25 ± 1.09 to 4.69 ± 0.22 ng/mL. These results indicate that the incorporation of either antimicrobial agent or ocular dye does not disrupt the *in vitro* release profile of rhNGF from the loaded lenticules (Figure 4).

### Slit lamp evaluation

**FIGURE 5:**
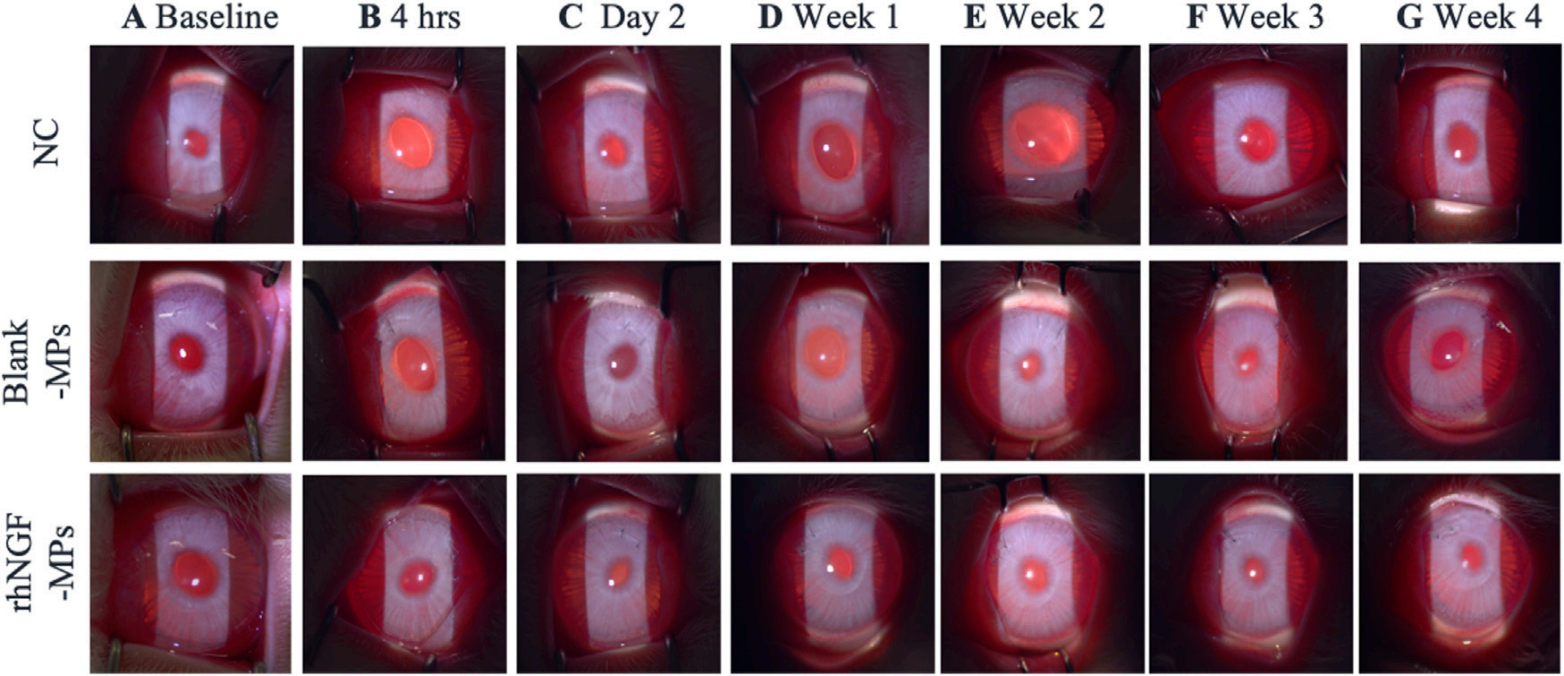
Representative slit lamp photographs showing the lenticule implantation area at **(A)** baseline, **(B)** 4hrs, **(C)** day 2, and **(D)** 1 week **(E)** 2 weeks **(F)** 3 weeks and **(G)** 4 weeks after surgical procedure. NC = negative control (untreated control), blank-MPs = implanted lenticules loaded with blank microparticles, rhNGF-MPs = implanted lenticules with microparticles loaded with recombinant human nerve growth factor. No signs of corneal haze, edema or infiltration was observed around the implantation site throughout the entire follow-up duration up to 1 month.

On slit lamp evaluation, the lenticules were well centered. No sign of corneal haze, inflammation, edema or infiltrate was observed at the site of implantation at any time point across all groups (Figures 5A–G), showing the clinical biocompatibility of using SMILE-derived decellularized lenticules as a non-immunogenic delivery system for loading therapeutic drugs.

### Anterior segment optical coherence tomography (ASOCT) evaluation

**FIGURE 6:**
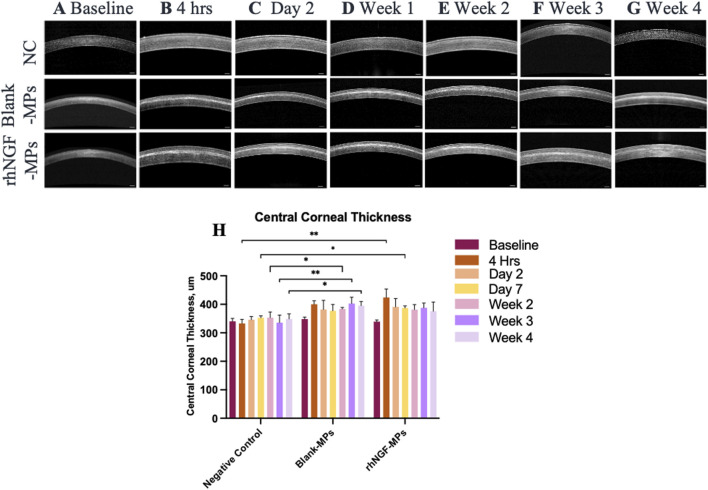
Representative ASOCT images depicting the central cornea thickness (CCT) at **(A)** baseline (pre-implantation), **(B)** 4 h, **(C)** day 2, **(D)** 1-week, **(E)** 2-week, **(F)** 3-week, and **(G)** 4-week after lenticule implantation. **(H)** Quantification analysis of CCT at respective time points (n = 3 images/time point). Mann-Whitney U statistical analysis was carried out with respective to the NC. *P ≤ 0.05, **P ≤ 0.01, ***P ≤ 0.001. Scale bar, 250 μm.

The site of lenticule implantation was further examined using ASOCT. As seen in Figures 6A–G, lenticules loaded with blank-MPs and rhNGF-MPs are consistently implanted at the corneal stroma layer. The central corneal thickness (CCT) of lenticule implantation groups were measured to be significantly higher when in comparison to the untreated NC group (Figure 6H). The CCT, either in the blank (401.45 ± 12.28 µm) or rhNGF group (421.55 ± 28.20 µm), increased in thickness of approximately 72.81 and 116.16 μm, respectively at 4 h after implantation as the lenticule thickness was measured maximum at 129 μm. It is noted that the measured CCT from either blank-MPs or rhNGF-MPs implant groups remained to be thicker throughout the 4 weeks follow-up when compared to its baseline (Figure 6H, Table 1). When comparing the CCT between blank-MPs and rhNGF-MPs groups, no statistically significant difference was noticed (Table 1), suggesting the increased CCT is likely to be from the lenticule thickness.

**TABLE 1:** CCT measurement of rabbit cornea implanted with lenticules loaded with blank-MPs and rhNGF-MPs at respective time points post-surgical procedure. All data show the normalized value with respective to baseline and set NC as 1 for comparison. *P* value >0.05 is considered non-significant (ns) and *p < 0.05 is considered significant.

Time point	NC	Blank-MPs	rhNGF-MPs	*p*-value^a^	Sig. Level
Baseline	1.00 ± 0.00	1.02 ± 0.02	1.00 ± 0.02	0.384	ns
4 h	1.00 ± 0.00	1.20 ± 0.04	1.26 ± 0.09	0.267	ns
Day 2	1.00 ± 0.00	1.10 ± 0.09	1.14 ± 0.08	>0.999	ns
Day 7	1.00 ± 0.00	1.07 ± 0.06	1.10 ± 0.05	0.888	ns
Week 2	1.00 ± 0.00	1.09 ± 0.03	1.08 ± 0.05	>0.999	ns
Week 3	1.00 ± 0.00	1.20 ± 0.06	1.15 ± 0.05	0.427	ns
Week 4	1.00 ± 0.00	1.13 ± 0.04	1.09 ± 0.08	0.361	ns

^a^ Comparisons between the blank-MPs, group and the rhNGF-MPs, group.

### *In vivo* confocal microscopy (IVCM) evaluation

**FIGURE 7:**
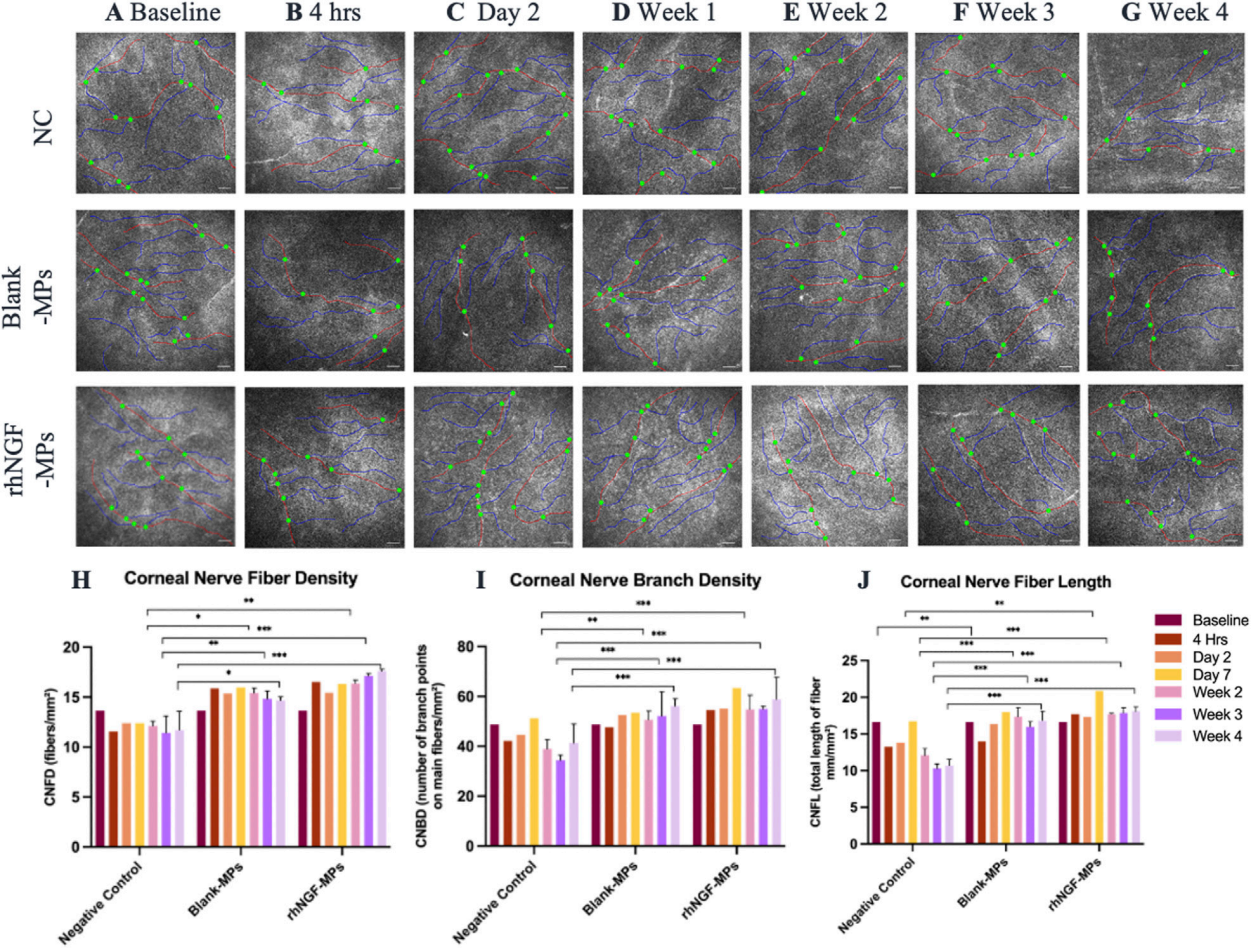
Representative *in vivo* confocal microscopy images taken from each group at **(A)** baseline (pre-implantation), **(B)** 4 h, **(C)** day 2, **(D)** week 1, **(E)** week 2, **(F)** week 3, and **(G)** week 4 after lenticule implantation. The potential of corneal nerve regeneration upon lenticule implantation was evaluated using CCMetrics software (University of Manchester, Manchester, UK) up to 1 month follow-up by assessing three corneal nerve parameters: **(H)** corneal nerve fiber density (CNFD; fibers/mm^2^), **(I)** corneal nerve branch density (CNBD; number of branch points on main fibers/mm^2^), **(J)** corneal nerve fiber length (CNFL; total length of fiber mm/mm^2^). Nerve fibres are marked by red lines, nerve branches are marked by blue lines, and branch points are marked by green dots. Mann Whitney statistical analysis was carried out with respective to the respective time point of NC group. *P ≤ 0.05, **P ≤ 0.01, ***P ≤ 0.001 (n = 10-15 images/group/time point). Scale bar, 100 μm.

The potential stimulating effects on corneal nerve from the *in vivo* release of rhNGF-MPs from engineered lenticule was assessed by the IVCM images taken at each follow-up time point. (Figures 7A–G). This observation was supported by the quantification analysis using CCMetrics, showing an increase detection of CNFD, CNBD, and CNFL at all time points when comparing between the treated (blank-MPs and rhNGF-MPs) and untreated NC group (Figures 7H–J). Specifically, a significant increase in all three corneal nerve parameters was present at week 2, week 3, and week 4 post-implantation from bioengineered lenticule treated (blank-MPs and rhNGF-MPs) with respect to untreated NC group (***p < 0.001; Figures 7H–J).

For better comparison, the computed values of all three corneal nerve parameters at each time point were normalized to its pre-implantation baseline, followed by normalizing to untreated NC group set as 1. To further evaluate the potential stimulating effect of the released *in vivo* rhNGF on corneal nerves, we compared statistical differences on all three corneal nerve parameters measured in rhNGF-MPs implant and blank-MPs group at all time points (see Table 2). Our results showed that CNFD increased significantly at 3 weeks in the lenticule implantation group loaded with rhNGF-MPs (1.50 ± 0.25-fold (*p* = 0.041)) as compared to the blank-MP group (1.28 ± 0.16-fold), and the increase of CNFD measured in rhNGF-MPs group became even more significantly apparent at the end of week 4 (blank-MPs group: 1.19 ± 0.19-fold; rhNGF-MPs group: 1.54 ± 0.14-fold (*p* ≤ 0.001)). On the other hand, a slight increase in CNFL was detected at day 7 post-implantation in rhNGF-MPs group (1.25 ± 0.07-fold (*p =* 0.027)) as compared to that of in blank-MPs group (1.08 ± 0.14-fold); the continuous increase in both groups up to 4 weeks follow-up were however, not significantly different (Table 2). The CNBD showed comparable measurements without any significant differences at all time points.

**TABLE 2:** Quantification analyses of corneal nerves using CCMetrics software from *in vivo* confocal microscopy images taken before (baseline) and after lenticule implantation procedures at respective time points (4 h, day 2, day 7, week 2, week 3, and week 4).

Time point	NC	Blank-MPs	rhNGF-MPs	*p*-value^a^	Sig. level
Corneal nerve fiber density (CNFD; fibers/mm^2^)					
Baseline	1.00 ± 0.00	1.00 ± 0.00	1.00 ± 0.00	—	—
Day 2	1.00 ± 0.00	1.24 ± 0.23	1.25 ± 0.14	>0.999	ns
Day 7	1.00 ± 0.00	1.29 ± 0.27	1.32 ± 0.24	>0.999	ns
Week 2	1.00 ± 0.00	1.28 ± 0.28	1.35 ± 0.28	>0.999	ns
Week 3	1.00 ± 0.00	1.28 ± 0.16	1.50 ± 0.25	0.041	*
Week 4	1.00 ± 0.00	1.19 ± 0.19	1.54 ± 0.14	0.001	***
Corneal nerve branch density (CNBD; no. of branch points on main fibers/mm^2^)					
Baseline	1.00 ± 0.00	1.00 ± 0.00	1.00 ± 0.00	—	—
Day 2	1.00 ± 0.00	1.13 ± 0.23	1.23 ± 0.21	>0.999	ns
Day 7	1.00 ± 0.00	1.04 ± 0.26	1.24 ± 0.20	>0.999	ns
Week 2	1.00 ± 0.00	1.38 ± 0.23	1.45 ± 0.31	0.658	ns
Week 3	1.00 ± 0.00	1.56 ± 0.34	1.60 ± 0.16	>0.999	ns
Week 4	1.00 ± 0.00	1.37 ± 0.15	1.43 ± 0.27	>0.999	ns
Corneal nerve fiber length (CNFL; total length of fibers, mm/mm^2^)					
Baseline	1.00 ± 0.00	1.00 ± 0.00	1.00 ± 0.00	—	—
Day 2	1.00 ± 0.00	1.18 ± 0.12	1.26 ± 0.21	0.705	ns
Day 7	1.00 ± 0.00	1.08 ± 0.14	1.25 ± 0.07	0.027	*
Week 2	1.00 ± 0.00	1.46 ± 0.21	1.47 ± 0.16	>0.999	ns
Week 3	1.00 ± 0.00	1.58 ± 0.17	1.75 ± 0.16	0.082	ns
Week 4	1.00 ± 0.00	1.58 ± 0.20	1.69 ± 0.18	0.484	ns

^a^ Comparisons between the blank-MPs group and the rhNGF-MPs group. All data show the normalized value with respect to the baseline, and set NC as 1 for comparison. *P* value >0.05 is considered non-significant (ns), *p ≤ 0.05 is considered significant, **P ≤ 0.01, ***P ≤ 0.001.

**FIGURE 8:**
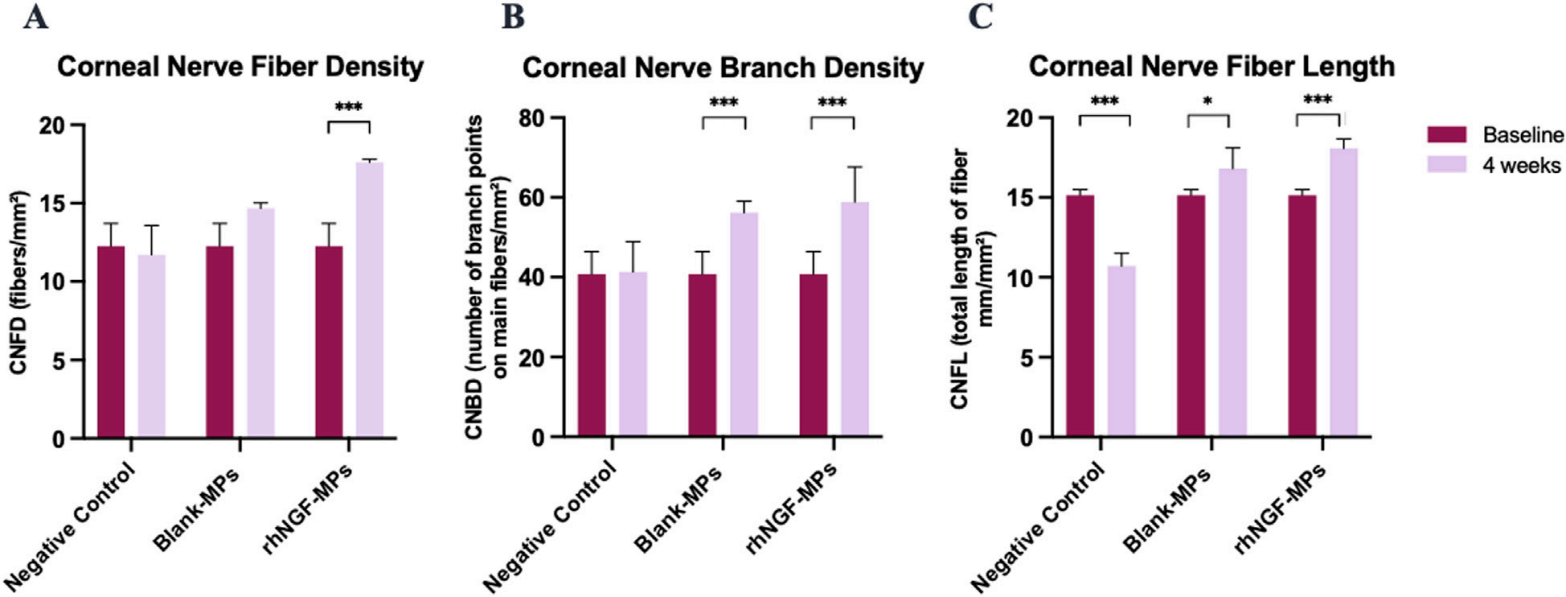
Bar charts showing the effect of lenticule implantation loaded with rhNGF-MPs on **(A)** CNFD, **(B)** CNBD, and **(C)** CNFL at 4 weeks follow-up when in comparison to the measurement performed at baseline in respective group. *p ≤ 0.05 is considered significant, **P ≤ 0.01, ***P ≤ 0.001.

Further evaluation of the potential stimulating effect of *in vivo* rhNGF release from bioengineered decellularized lenticule on corneal nerve was conducted by comparing CNFD, CNBD, and CNFL obtained at 4 weeks post-implantation to the pre-implantation baseline measurement. Our results showed that CNBD and CNFL were significantly increased after 4 weeks of lenticule implantation loaded with either blank-MPs (CNBD: 56.63 ± 6.47 branch points on main fibers/mm^2^ (***p < 0.001); CNFL: 16.98 ± 2.22 mm/mm^2^ (*p = 0.026)) or rhNGF-MPs (CNBD: 58.98 ± 11.31 branch points on main fibers/mm^2^ (***p < 0.001); CNFL: 18.09 ± 2.03 mm/mm^2^ (***p < 0.001)) (Figures 8B,C, Table 3) as compared to the baseline measurement (CNBD: 40.66 ± 10.52 branch points on main fibers/mm^2^; CNFL: 15.06 ± 2.22 mm/mm^2^), whereas the increase in CNFD was only observed in the rhNGF-MPs implant group (week 4: 17.61 ± 2.23 fibers/mm^2^ (***p < 0.001); baseline: 13.16 ± 3.95 fibers/mm^2^).

**TABLE 3:** Evaluation of the corneal nerve regeneration potential at 4-week after lenticule implantation loaded with rhNGF-MPs.

Time point	NC	Blank-MPs	rhNGF-MPs
Corneal nerve fiber density (CNFD; fibers/mm^2^)			
Baseline	13.16 ± 3.95	13.16 ± 3.95	13.16 ± 3.95
Week 4	11.90 ± 3.47	14.67 ± 2.01	17.61 ± 2.23
*p*-value^a^	0.287	0.056	<0.001
Sig. level	ns	ns	***
Corneal nerve branch density (CNBD; no. of branch points on main fibers/mm^2^)			
Baseline	40.66 ± 10.52	40.66 ± 10.52	40.66 ± 10.52
Week 4	41.32 ± 8.94	56.63 ± 6.47	58.98 ± 11.31
*p*-value^a^	0.732	<0.001	<0.001
Sig. level	ns	***	***
Corneal nerve fiber length (CNFL; total length of fibers, mm/mm^2^)			
Baseline	15.06 ± 2.22	15.06 ± 2.22	15.06 ± 2.22
Week 4	10.71 ± 1.49	16.98 ± 2.22	18.09 ± 2.03
*p*-value^a^	<0.001	0.026	<0.001
Sig. level	***	*	***

^a^ Comparisons between the measurement performed at week 4 and baseline. *P* value >0.05 is considered non-significant (ns), *p ≤ 0.05 is considered significant, **P ≤ 0.01, ***P ≤ 0.001.

### *In vivo* release of rhNGF in rabbit model

**FIGURE 9:**
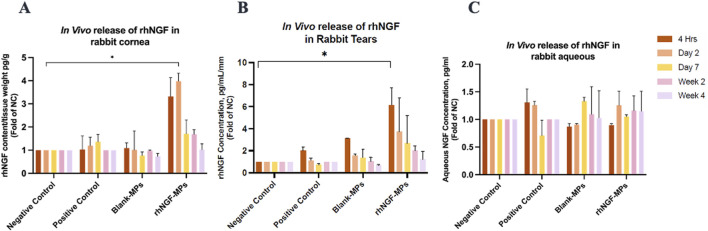
Bar charts showing the NGF levels in different rabbit samples. *In vivo* rhNGF contents detected in rabbit **(A)** corneas, **(B)** tears, and **(C)** aqueous humour at 4 h, day 2, day 7, week 2, and week 4 post-implantation in respective groups. All data was normalized to endogenous NGF concentration in NC group. *P ≤ 0.05.

The experiments of engineering decellularized lenticules with rhNGF-MPs were performed in the identical way as for *in vitro* experiments. The kinetic *in vivo* of rhNGF release from the engineered decellularized lenticules were evaluated by collecting rabbit cornea, tears and aqueous humour at 4 h, day 2, every week until up to 1-month after implantation (Figure 9). As seen in Figure 9A, NGF content in rabbit cornea from rhNGF-MPs implant group is significantly higher (3.98 ± 0.34-fold (*p = 0.035)) than that of the endogenous NC group at day 2 after implantation) (Table 4). Of note, a gradually reduced *in vivo* release of rhNGF was detected in rabbit cornea along the progression of rhNGF-MPs implant treatment, and eventually returned to be at comparable level as the endogenous NC group. It is worth mentioning that the rhNGF content detected in rabbit cornea still remained to be higher than all the other groups at all time points, although the differences were not statistically significant. Nevertheless, these results suggested a rapid release in rabbit corneas in the first 2 days and maintained a slow release of rhNGF for up to 1-month *in vivo* (Table 4, Figure 9A).

**TABLE 4:** *In vivo* release profile of rhNGF detected from rabbit cornea, rabbit tears and rabbit aqueous after lenticules implantation loaded with rhNGF-MPs at different time points.

Time point	NC	PC	*p*-value^a^	Blank-MPs	*p*-value^b^	rhNGF-MPs	*p*-value^c^	*Sig. level*
NGF contents (pg/g) in rabbit cornea								
4 h	1.00	1.03 ± 0.59	>0.999	1.09 ± 0.23	>0.999	3.32 ± 0.81	0.064	ns
Day 2	1.00	1.20 ± 0.36	>0.999	1.01 ± 0.83	>0.999	3.98 ± 0.34	0.035	*
Day 7	1.00	1.36 ± 0.31	0.523	0.77 ± 0.15	0.925	1.71 ± 0.60	0.268	ns
Week 2	1.00	1.00 ± 0.00	>0.999	0.97 ± 0.02	>0.999	1.69 ± 0.19	0.147	ns
Week 4	1.00	1.00 ± 0.00	>0.999	0.73 ± 0.12	0.742	1.02 ± 0.26	>0.999	ns
Tear NGF concentration (pg/mL/mm)								
4 h	1.00	2.00 ± 0.35	>0.999	3.11 ± 0.34	0.307	6.15 ± 1.54	0.043	*
Day 2	1.00	1.12 ± 0.20	>0.999	1.56 ± 0.18	0.771	3.74 ± 2.50	0.392	ns
Day 7	1.00	0.73 ± 0.12	0.307	1.37 ± 0.55	>0.999	2.66 ± 1.83	>0.999	ns
Week 2	1.00	1.00 ± 0.00	0.965	1.04 ± 0.36	>0.999	2.01 ± 0.43	0.147	ns
Week 4	1.00	1.00 ± 0.00	0.965	0.66 ± 0.08	0.527	1.24 ± 0.70	>0.999	ns
Aqueous NGF concentration (pg/mL)								
4 h	1.00	1.31 ± 0.25	>0.999	0.87 ± 0.06	0.459	0.90 ± 0.03	0.922	ns
Day 2	1.00	1.26 ± 0.07	0.662	0.91 ± 0.01	>0.999	1.26 ± 0.25	0.662	ns
Day 7	1.00	0.71 ± 0.28	0.922	1.33 ± 0.07	0.922	1.05 ± 0.03	>0.999	ns
Week 2	1.00	1.00 ± 0.00	>0.999	1.10 ± 0.50	>0.999	1.16 ± 0.27	>0.999	ns
Week 4	1.00	1.00 ± 0.00	>0.999	1.03 ± 0.49	>0.999	1.15 ± 0.36	>0.999	ns

^a^ Comparisons between the NC, group and PC, group.

^b^ Comparisons between the NC, group and blank-MPs, group.

^c^ Comparisons between the NC, group and rhNGF-MPs, group.

*P* value >0.05 is considered non-significant (ns), *p ≤ 0.05 is considered significant, **P ≤ 0.01, ***P ≤ 0.001.

In comparison to the *in vivo* release results in cornea, a similar pattern was observed in rabbit tears of rhNGF-MPs group. A significantly higher tear NGF concentration/mm was detected than that in the control group (Figure 9B) at 4 h time point (6.15 ± 1.54-fold (*p = 0.043)), and gradually decreased over time. In contrast to the results obtained in rabbit corneas and tears, there was no significant difference in the rabbit aqueous sample in all groups across all time points (All *p* > 0.05; Figure 9C).

## Discussion

Drug delivery systems for corneal repair include the use of amniotic membrane (AM) (Wan et al., 2021), exosomes (Robbins et al., 2024), biopolymers (Zarrintaj et al., 2018), and bioengineered lenticules derived from SMILE procedures (Mastropasqua et al., 2022). While AM transplantation delivering rhNGF was reported to promote corneal epithelium and corneal nerve regeneration in rabbit model with corneal epithelial defect (Wan et al., 2021), exosomes have been used as novel carriers for cornea-targeted gene therapy or biologic therapeutics owing to their inherent function in intracellular communication and biocompatibility (Burnouf et al., 2019). However, optimization for maximum threshold, stability and storage of rhNGF-AM, as well as the wound-healing effect, dosage, frequency and cost of medication (Wan et al., 2021), are necessary to be carefully evaluated for its clinical therapeutic efficiency. On the other hand, the success of exosome therapy is highly dependent on several technical obstacles such as tedious exosome preparations and validation, limited drug loading capacity, and require more work to address their storage stability, *in vivo* performance and clinical efficacy (Palakurthi et al., 2024; Bhujel et al., 2023). Similarly, continuous challenges remain in biopolymer-based drug delivery system to find balance between better corneal penetrations and longer drug retention time with minimal cytotoxicity or discomfort (Kim et al., 2020).

SMILE, available with the VisuMax FS was first clinically introduced in Europe by Sekundo et al. (2011) is currently the most common keratorefractive lenticule extraction (KLEx) procedures. During the SMILE procedure, a thin piece of intrastromal lenticule created by femtosecond laser for refractive correction is extracted through a small arcuate incision as a by-product, and is usually discarded (Yu Chi et al., 2017). Numerous advantages of using SMILE-derived lenticules include its low immunogenicity, possibility to cryo-store without affecting the optical and biomechanical characteristics (Liu et al., 2017b; Hori and Niederkorn, 2007; Lagali, 2020). In clinical ophthalmology, utilizing the naturally present collagen-rich extracellular matrix (ECM) in corneal stroma as scaffold has offered a new perspective on corneal regenerative medicine (Santra et al., 2022). It is more favored compared to other biosynthetic materials for directing tissue morphogenesis, differentiation and homeostasis in a more native physiological manner (Mobaraki et al., 2019; Santra et al., 2022). In addition to SMILE, other KLEx procedures, including corneal lenticule extraction for advanced refractive correction (CLEAR; Ziemer), smooth incision lenticule keratomileusis (SILK; Johnson & Johnson), and small incision–guided human cornea treatment (SmartSight; SCHWIND eye-techsolutions), have also emerged. Hence, the source of lenticules for bioengineering can come from any of the KLEx procedures.

Over the years, SMILE-derived lenticules have been repurposed for the treatment of a variety of ocular diseases in several clinical studies. Besides being applied as a patch graft for corneal defect (Bhandari et al., 2016), lenticule transplantation into stromal layer has also been established for the treatment of keratoconus, hyperopia, presbyopia without postoperative complications, such as corneal haze or immune rejection (Ganesh and Brar, 2015; Sun et al., 2015; Liu et al., 2018; Riau et al., 2020). A recent study from our group has demonstrated the re-innervation potential of decellularized human lenticules upon stimulation of the grafted chick dorsal root ganglion model supplemented with NGF (Yam et al., 2022), suggesting tremendous capacity of using decellularized human lenticules for treatment of ocular diseases.

While topical ophthalmic eye drop remains as conventional therapy for the treatment of most ocular diseases owing to its noninvasiveness, ease of administration, and low cost, the therapeutic efficacy is still restricted by the low bioavailability and poor patient compliance (Pflugfelder et al., 2020; Jumelle et al., 2020; Liu et al., 2020). The biocompatible SMILE-derived lenticule as ocular drug delivery system has a superior feature to load different drugs of interest and has the capacity to fine tune the drug release profiles. For example, adjustment of the nanocarrier formulation or drug loading concentrations could implicate the targeted therapeutic efficiency. Encapsulating drugs in FDA-approved PLGA nanoparticles has been commonly adopted in the field of tissue engineering and drug delivery to improve drug release kinetics with sustained release profile (Gupta et al., 2010; Bhosale and Devi, 2012). In this regard, Mastropasqua and colleagues have circumvented the eye drop limitation by exploring the potential application of using SMILE-derived lenticule to deliver pharmaceutically active molecule for ocular disease treatment (Mastropasqua et al., 2022). In particular, rhNGF loaded PLGA-MPs (rhNGF-MPs) was successfully incorporated both on the lenticule surface and its stroma of the decellularized human lenticules with an encapsulation efficiency of 79% (Mastropasqua et al., 2022). The *in vitro* release kinetic showed a sustained release for up to 1 month of rhNGF from MPs loaded to the lenticule, with approximately 80% preservation of rhNGF activity in an *in vitro* model using PC12-Luci cells (Mastropasqua et al., 2022).

On this basis, this study aimed to validate the *in vitro* release, followed by further investigating the *in vivo* release of rhNGF using a rabbit model. Our *in vitro* release profile was in consensus with the previous finding (Mastropasqua et al., 2022), in the way that we have also observed initial burst release of rhNGF and then it was sustained up to the end of experiment of 7 days (Figure 2). The similar *in vitro* release profile has demonstrated the reproducibility of the experimental design. The variation of the amount of cumulative release of rhNGF from the rhNGF-MPs loaded lenticules observed between our study (4.84 ± 0.12 ng/mL at day 7) and the previous study (approximately 11 ng/mL at day 7) (Mastropasqua et al., 2022) could be due to different source of lenticules and batches of rhNGF-MPs used. Although the % total release *in vitro* at day 3 and day 7 appeared to be low, it is important to note that the detected *in vitro* rhNGF concentration (0.2584 ng/mL and 0.1425 ng/mL, respectively) are substantially higher than the endogenous NGF levels measured in rabbit cornea ranging from 27 to 65 pg/mL in this study. Besides, our previous study demonstrated that the biological activity of the released rhNGF is preserved at 70% and 81% relative to the rhNGF reference standard at concentrations of 0.3750 ng/mL and 0.1875 ng/mL, respectively (Mastropasqua et al., 2022). These findings indicate that despite modest % total release *in vitro* from day 2 onwards, the released rhNGF maintains biologically active and exceeds the physiological levels in our testing model. Moving forward, we have extended the investigation to rabbit model using the bioengineered SMILE-derived lenticules from human cadaveric corneas to further examine the *in vivo* sustained release profile of rhNGF, potential pathological changes, as well as the nerve regeneration capacity upon re-implantation of rhNGF-MPs loaded lenticules.

Upon lenticule implantation, we have first demonstrated that rhNGF-MPs loaded lenticules are biocompatible by showing no sign of corneal inflammation, edema, or infiltration at the site of implantation throughout the entire follow-up procedures (Figures 5A–G). While analyzing the results from ASOCT and IVCM data, we have observed inter-animal baseline variability due to inherent differences. Although the variability seen was not apparent, it is important to address it adequately to minimize the negative impact on actual therapeutic effects and data reliability. To evaluate the actual therapeutic effects of rhNGF, the measurements at each time point obtained from ASOCT and IVCM were first normalized to its pre-implantation baseline, followed by normalizing to untreated NC group set as 1. As such, the significant differences observed in our studies were accounted for the true effects that were attributed by the rhNGF relative to the untreated group. A general increase in CCT was seen in both blank-MPs and rhNGF-MPs implant group ranging from 72.81 to 116.16 μm at 4 h after implantation. This was due to the lenticule thickness we implanted. The successful and consistent lenticule implantation procedure without pathological observation in slit lamp or ASOCT further substantiates the biocompatibility of bioengineered human stromal lenticule. NGF, a neurotrophic factor, is known to promote neuroprotection and neurogenesis (Rizzi et al., 2018; Quarta et al., 2014). Corneal nerve analysis showed significant increase in CNFD and CNFL in rhNGF-MPs implant group when in comparison to blank-MPs group (Table 2). The corneal nerve regenerative effects are likely to be mediated by rhNGF through TrkA signaling (De Castro et al., 1998). The NGF-activated TrkA signaling pathway could subsequently trigger several signaling pathways including PLC-γ1, MAPK, and PI3K (Gupta et al., 2022). The NGF-bound TrkA, along with its associated signaling intermediates are collectively essential for cell survival, neurite outgrowth, neuronal differentiation, as well as specification and synapse formation of sensory and sympathetic neurons (Gupta et al., 2022; Harrington and Ginty, 2013). The other possible explanation might be attributed by the NGF effects in accelerating autophagic clearance of myelin debris in Schwann cells (SC), which was hypothesized to be regulated by the p75^NTR^ AMPK/mTOR axis (Li et al., 2020). In the event of nerve injury, degradation of myelin debris by SC during the first 5–7 days has been reported to be a prerequisite for nerve regeneration (Perry et al., 1995). With the treatment of NGF, myelin debris clearance was activated, and was found to be associated with an increased activity of autophagy of SC in a nerve crush injury *in vivo* model, thereby contributing to early nerve regeneration (Li et al., 2020). The observation of significant increase in CNBD and CNFL after 4 weeks of lenticule implantation loaded with and without rhNGF-MPs (Figures 8B,C, Table 3) could be attributed by the lenticule implantation process. When comparing among different time points, all three corneal nerve parameters CNFD, CNBD, and CNFL from the rhNGF-MPs implant group were measured to be significantly higher at week 4 than the baseline measurement (Table 3), implicating its great potential on enhancing nerve regeneration from released rhNGF *in vivo* on corneal nerves. Moreover, the CNFD showed a significantly differential increase in fiber density than branch density after 4 weeks of rhNGF treatment, indicating that the corneal nerve regeneration observed was contributed more by main nerve regeneration than branch regeneration. Our positive results are in line with one of our previous clinical studies in which the mean nerve density in rhNGF-eye drops treated NK patients showed a significant increase (*p* = 0.007) after 8 weeks of treatment (Mastropasqua et al., 2020). This consensual results were further substantiated by similar results observed in a recent clinical study (Hao et al., 2023). Establishing a lenticule banking system would therefore be useful to reserve for future therapeutic needs (Riau et al., 2022). The feasibility for experimental uses was validated by implanting short- (3-month) or long-term (12-month) cryopreserved lenticule into rabbit corneal stroma (Riau et al., 2022). Results showed maintained corneal morphology, with no active wound healing and inflammatory reactions even at 16-week post-implantation, suggesting the feasibility of using long-term lenticule banking for clinical therapeutics.

To account for potential confounding factors, such as surgical intervention and the innate healing response unrelated to rhNGF delivery, we included positive control groups where rabbits underwent pocket creation without lenticule implantation. The NGF contents detected in rabbit cornea, tears and aqueous shown in positive control group (Figures 9A–C) represent the innate healing response associated with pocket creation and stitching procedures. Additionally, to strengthen the analysis of rhNGF’s therapeutic effects *in vivo*, we evaluated the *in vivo* rhNGF release from the Blank-MPs group subjected to re-implantation of SMILE-derived lenticules loaded with blank MPs, simulating the innate healing response from lenticule re-implantation in conjunction with pocket creation. As seen in Figures 9A–C, corneal and tear NGF content in the PC and Blank-MPs groups are comparable to the NC group without any surgical intervention, indicating that the significantly higher NGF levels observed in the experimental group (rhNGF-MPs) are primarily due to rhNGF release from lenticule re-implantation, with minimal confounding effects from surgical trauma or innate healing responses. For future clinical applications, other potential confounding factors, such as age, ocular surface conditions, previous ocular surgical trauma, and medical history, must be carefully considered to avoid masking the effects of rhNGF when evaluating the effectiveness of bioengineered rhNGF delivery using SMILE-derived lenticules.

Our data has shown, for the first time, that the loaded rhNGF-MPs were able to sustain in the bioengineered lenticule *in vivo* for at least 2 weeks with 3.32-fold higher NGF content detected in rabbit cornea at 4 h (*p* > 0.05), 3.98-fold higher at day 2 (*p* = 0.035), 1.71-fold higher at week 1 (*p* > 0.05), and 1.69-fold higher at week 2 (*p* > 0.05), when compared to NC group without undergoing any surgery (Figure 9A; Table 4) before returning to be comparable to the NC group at week 4 (1.02-fold, p > 0.05). It is worth indicating that the obtained results were from two separate batches of implantation on a separate day, both with consistent finding of a sustained release detected until the end of the experiment of 4 weeks *in vivo*, highlighting its promising potential for future clinical application. Interestingly, a similar pattern was observed in rabbit tears of rhNGF-MPs group, with a higher tear NGF concentration detected at all time points after implantation than in endogenous tear NGF. However, only the tear NGF detected at 4 h post-implantation were noted to be significantly higher than that of the NC group. Nevertheless, repeating experiments using more sample sizes should be carried out to better elucidate the potential association of NGF contents detected in cornea and tears upon implantation. On the contrary, the NGF content detected in aqueous humour showed comparable level across all groups (*p* > 0.05; Figure 9C), indicating that the rhNGF release localized in the corneas and did not reach the anterior chambers even after 1 month.

It is worth pointing out that our study has also employed the decellularized lenticules for both *in vitro* validation and *in vivo* experiments. Despite the immune privilege of corneal stroma tissue, immune rejection and inflammation can still occur following allogenic lenticule implantation (Hashimoto et al., 2016). Decellularization, on the other hand, can further reduce the potential risk of stromal immune rejection, thereby improving its allogeneic clinical applications. Moreover, employing decellularization procedure would also enable a simpler, and more accurate *in vitro* release analysis without the necessity to consider the intrinsic NGF in the case of adopting a non-decellularized lenticule. The space created by decellularization could also potentially enhance the loading efficiency of therapeutic substances.

Inlay implantation undergoes a very similar procedure to the implantation of bioengineered lenticules with therapeutic substances, where a synthetic material is permanently inserted within the corneal stromal layer. In cases of intracorneal ring implantation (Microbiology and Diseases) for refractive surgery (Schanzlin et al., 2001), post-laser *in situ* keratomileusis ectasia (Siganos C. et al., 2002), or keratoconus (Siganos D. et al., 2002), the incidence of infectious keratitis following ICR has been documented ranging from 1.4%–6.8% (Mulet et al., 2010; Tabatabaei et al., 2019; Ferrer et al., 2010). Although microbial keratitis is not a common complication, the subsequent pathological manifestation could be devastating and even cause loss of vision. Appropriate measure should therefore be taken to either minimize the occurrence or rescue it from developing further complications which are sight-threatening. For this reason, we have incorporated the antimicrobial agents into the washing steps of lenticule preparation and performed *in vitro* release to ensure the compatibility with rhNGF. As seen in Figure 4, the incorporation of antimicrobial agents did not seem to impede the release of rhNGF over time, which promotes its potential application, particularly penicillin, streptomycin and amphotericin B as a consensus measure to minimize the incidence of microbial keratitis from bioengineered lenticule implantation.

On the other hand, a potential surgical challenge for lenticule implantation could be contributed by the intrinsic property of corneal stromal being highly transparent (Zhang et al., 2017). In this regard, we think that a short treatment using commonly used ophthalmic dye such as trypan blue and fluorescein could offer great help, particularly for surgeons at the beginner level or especially in the clinical setting equip with limited accessibility to microscope with high resolution. Our *in vitro* release data showed comparable profile in rhNGF-loaded lenticules treated with or without ophthalmic dyes, both displaying increasing cumulative rhNGF release over time (Figure 4). These positive observations suggest the suitability of adopting trypan blue or fluorescein as visual enhancement tools for ophthalmic surgeons to better position the therapeutic loaded lenticules during the re-implantation procedures.

One limitation in our study is the occasional observation of lenticule shrinkage after dehydration at 60 °C for 2 h, ranging from −3.27% to −51.99% (Supplementary Figures 1A, B). The shrinkage of the lenticules remain upon rehydration in rhNGF suspension for *in vitro* or *in vivo* release assessments (Figures 3A,B) which might account for the differences in the *in vitro* release results observed in this study compared to the prior work. This observation might be due to the SMILE’s setting of minimal thickness set at 15 μ m peripheral edge. Lenticule with thinner peripheral edge holds lesser volumetric structure and could be more susceptible to drastic lenticule shrinkage after dehydration. Indeed, we found that the lenticle shrinkage became less noticeable (−3.36%, −7.85%; Supplementary Figures 1C, D) after adjusting the femtosecond laser parameter to obtain lenticules with thicker peripheral edge set at 30 μ m. To investigate further, we repeated the lenticule excision experiments using a total of 17 lenticules. As expected, the average lenticule shrinkage is trivial with only −4.01% (Supplementrary Figure 2), confirming our abovementioned hypothesis. The peripheral edge set at 30 μ m during lenticule preparation could be an important parameter as a standardized reference for future clinical studies. Despite the lenticule shrinkage observed at times, the phenomenon does not seem to affect the *in vitro* release profile, still showed expected sustained release profile over the course of experiment (Figure 4). Nevertheless, for clinical scalability, a standardized lenticule preparation, together with batch-to-batch variability of the rhNGF-MPs production and bioactivity should be carefully monitored with stringent control to ensure reproducible drug loading for its therapeutics.

Taken all together, our study has not only successfully validated the previously described procedures of lenticule preparation (Mastropasqua et al., 2022), the subsequent *in vivo* experiments using the same lenticule preparation method, exhibited significantly higher NGF content in rabbit cornea and tears compared to the endogenous NC group, proving that SMILE-derived human lenticules holds capacity to sustain rhNGF-MPs both *in vitro* for at least 7 days and *in vivo* for up to 1 month without any distinctive pathological changes. This underlies the potential for setting lenticule preparation as the standardized procedure for future donor’s lenticule preparation. It also creates more options for various ocular treatments which consider drug-loaded lenticule implantation as a therapeutic solution. Following the method validation *in vitro*, we have also showed the lenticule implantation loaded with rhNGF-MPs is feasible without post-surgical complications *in vivo*. In addition, the significant increase in CNFD after 3 weeks of rhNGF-MPs implantation are positive indication of corneal nerve re-innervation. The *in vivo* release profile of rhNGF provided us a better guidance on the subsequent treatment regimen planning to move forward to clinical studies. The implantation of such lenticules loaded with rhNGF-MPs, in corneas with stromal ulceration in NK, may theoretically combine the tectonic support and tissue replacement of the stromal supplementation (similarly to that obtained using amniotic membrane transplantation) with the pharmacological effect of the released NGF, in the recipient pathological corneas. The limitation of this study will be addressed in future studies by including the evaluation of corneal sensitivity and corneal epithelial analysis to garner a more comprehensive assessment on the bioactivity or functionality of rhNGF released from the scaffold *in vivo*. Extending the study to an animal model with corneal denervation will also provide more insights on to nerve regeneration potential for future clinical applications.

## Conclusion

In summary, this study has demonstrated a successful *in vivo* implantation of bioengineered lenticules with rhNGF with no sign of adverse effects on corneas. We have shown the desirable and sustained rhNGF *in vivo* release profiles from bioengineered lenticules for up to 1 month. The detection of least 1-month of rhNGF release *in vivo*, together with the early observation of significant increase in CNFD after 3 weeks treatment, have greatly depicted its translational potential for its clinical application in the near future. Besides, a pre-treatment on lenticules with antimicrobial agents on rhNGF-loaded lenticules without affecting the *in vitro* release profile of rhNGF imparts its potential clinical application to prevent inlay-related infection arising from surgical procedures. The comparable *in vitro* release profiles between groups treated with and without trypan blue or fluorescein offers the option of using trypan blue or fluorescein as visual assistive tool during the lenticule implantations.

## Data Availability

The original contributions presented in the study are included in the article/Supplementary Material, further inquiries can be directed to the corresponding authors.
