# Supplementary material for: Evaluation of in vitro and in vivo release of recombinant human nerve growth factor from bioengineered human stromal lenticule
Source: Front Bioeng Biotechnol. 2025 Sep 19;13:1588629. doi: 10.3389/fbioe.2025.1588629 (PMC12492493; doi:10.3389/fbioe.2025.1588629)
Supplement: Supplementary file 1 [file Supplementaryfile1.docx]

Supplementary Material


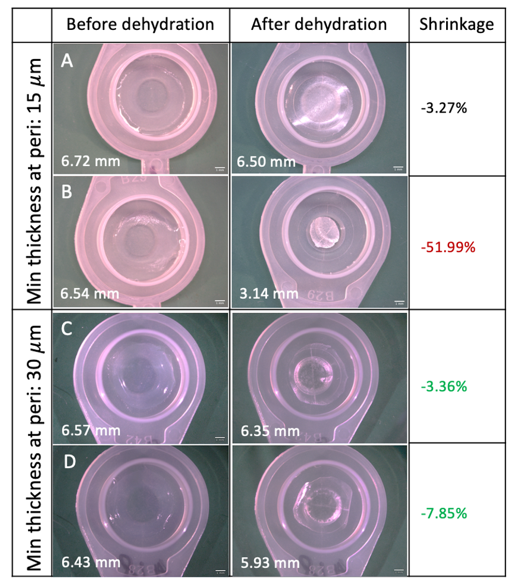


**Supplementary Figure 1.** Representative light microscopic images of lenticules taken before and after dehydration. (A) Lenticules with minimal thickness of 15 μm at peripheral edge showed 3.27% and (B) 51.99% shrinkage after dehydration at 60oC for 2h. (C) Lenticules with minimal thickness of 30 μm at peripheral edge has lesser lenticule shrinkage percentage at 3.36% and (D) 7.85% after dehydration.


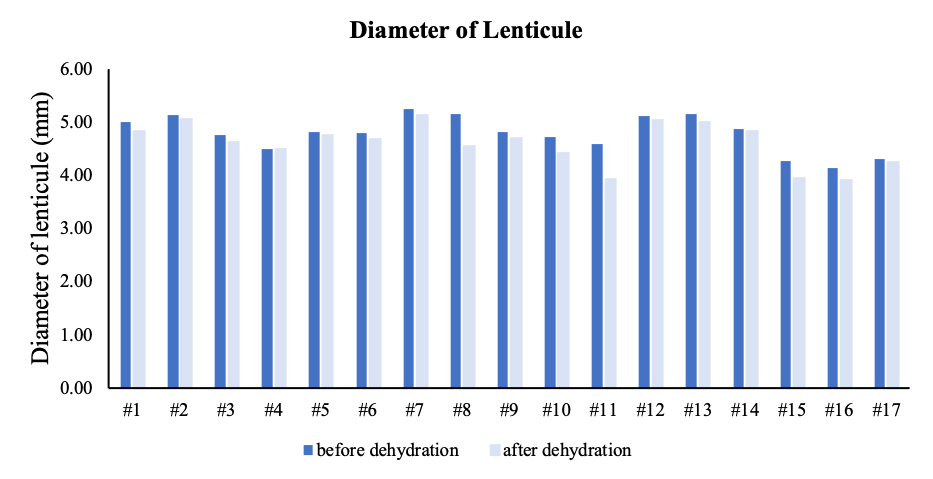


**Supplementary Figure 2.** Measurement of the diameter of lenticules at before and after dehydration (n=17 lenticules).
